# ROS-Dependent ER Stress and Autophagy Mediate the Anti-Tumor Effects of Tributyltin (IV) Ferulate in Colon Cancer Cells

**DOI:** 10.3390/ijms21218135

**Published:** 2020-10-30

**Authors:** Adriana Celesia, Ornella Morana, Tiziana Fiore, Claudia Pellerito, Antonella D’Anneo, Marianna Lauricella, Daniela Carlisi, Anna De Blasio, Giuseppe Calvaruso, Michela Giuliano, Sonia Emanuele

**Affiliations:** 1 Department of Biomedicine, Neurosciences and Advanced Diagnostics (BIND), Biochemistry Building, University of Palermo, Via del Vespro 129, 90127 Palermo, Italy; adriana.celesia@unipa.it (A.C.); marianna.lauricella@unipa.it (M.L.); daniela.carlisi@unipa.it (D.C.); 2 Laboratory of Biochemistry, Department of Biological, Chemical and Pharmaceutical Sciences and Technologies (STEBICEF), University of Palermo, Via del Vespro 129, 90127 Palermo, Italy; ornella.morana@gmail.com (O.M.); antonella.danneo@unipa.it (A.D.); anna.deblasio@unipa.it (A.D.B.); giuseppe.calvaruso@unipa.it (G.C.); 3 Department of Physics and Chemistry “Emilio Segrè” (DiFC), University of Palermo, Viale delle Scienze, Building 17, 90128 Palermo, Italy; tiziana.fiore@unipa.it (T.F.); claudia.pellerito@unipa.it (C.P.); 4 Inter-University Consortium for Research on the Chemistry of Metal Ions in Biological Systems (C.I.R.C.M.S.B.), Piazza Umberto I, 1-70121 Bari, Italy

**Keywords:** tributyltin (IV) derivative, ROS, oxidative stress, endoplasmic reticulum stress, autophagic cell death

## Abstract

Organotin compounds represent potential cancer therapeutics due to their pro-apoptotic action. We recently synthesized the novel organotin ferulic acid derivative tributyltin (IV) ferulate (TBT-F) and demonstrated that it displays anti-tumor properties in colon cancer cells related with autophagic cell death. The purpose of the present study was to elucidate the mechanism of TBT-F action in colon cancer cells. We specifically show that TBT-F-dependent autophagy is determined by a rapid generation of reactive oxygen species (ROS) and correlated with endoplasmic reticulum (ER) stress. TBT-F evoked nuclear factor erythroid-2 related factor 2 (Nrf2)-mediated antioxidant response and Nrf2 silencing by RNA interference markedly increased the anti-tumor efficacy of the compound. Moreover, as a consequence of ROS production, TBT-F increased the levels of glucose regulated protein 78 (Grp78) and C/EBP homologous protein (CHOP), two ER stress markers. Interestingly, Grp78 silencing produced significant decreasing effects on the levels of the autophagic proteins p62 and LC3-II, while only p62 decreased in CHOP-silenced cells. Taken together, these results indicate that ROS-dependent ER stress and autophagy play a major role in the TBT-F action mechanism in colon cancer cells and open a new perspective to consider the compound as a potential candidate for colon cancer treatment.

## 1. Introduction

Reactive oxygen species (ROS) represent a class of strongly reactive ions and molecules, which can be considered the physiological products of cell metabolism [1,2]. At low concentrations, ROS can act as signaling molecules involved in the regulation of a variety of biological processes [3]. It has been shown that cancer cells display increased ROS levels in comparison to their non-tumor counterparts, a condition which is partly due to enhanced cancer cell metabolism and mitochondrial dysfunction [4,5]. The increased ROS production in cancer cells contributes to the activation of biochemical pathways necessary for tumor progression and resistance to chemotherapy [6]. However, increased ROS levels in cancer cells may be considered a strategic opportunity to specifically target cancer cells by overcoming the tumor ROS threshold to highly toxic levels, thereby, stimulating ROS-dependent cell death pathways [7]. For this reason, increasing attention is focused on anticancer drugs that effectively kill cancer cells and overcome drug resistance by promoting ROS generation and/or affecting cellular antioxidant defense mechanisms [8].

As a response to oxidative stress, cells activate antioxidant defenses. Among these, nuclear factor erythroid-2 related factor 2 (Nrf2) represents a key player being involved in transcriptional activation of anti-oxidant genes [9,10]. Nrf2 function is negatively regulated by Keap1, which binds Nrf2 under physiological conditions and promotes its ubiquitination and consequent 26S proteasome-mediated degradation. Keap1 is a cysteine-rich protein and a ROS sensor that upon oxidative stress undergoes a conformational change that decreases affinity to Nrf2 and permits its nuclear translocation and transcriptional activity [11]. Nrf2 activation also depends on p62/sequestosome 1, simply known as p62, a multifunctional scaffold protein that can inhibit Keap1, thus promoting Nrf2 activation [12]. Since both Nrf2 and p62 result overexpressed in a number of tumors [13], targeting Nrf2/Keap1/p62 pathway can represent a tool to favor oxidative stress-dependent cell death in tumor cells [14,15].

It is well documented that ROS-mediated oxidative stress can evoke a cell response that involves physiological processes such as endoplasmic reticulum (ER) stress and autophagy [16].

ER stress is a well-known cellular process that results from physiological or pathological stresses including increased secretory load and accumulation of unfolded or misfolded proteins into the ER lumen. The accumulation of altered/misfolded proteins in the ER triggers an evolutionarily conserved program called the unfolded protein response (UPR) which is activated to free the ER from misfolded proteins and restore ER homeostasis [17]. On the other hand, UPR can generate ROS production supporting a mutual relationship between the two processes [18,19]. ER protein chaperones such as glucose regulated protein 78 (Grp78)/BiP finely regulate ER stress since they inhibit UPR sensors located in the ER membrane [20]. The sequestration of Grp78 by misfolded proteins results in the loss of repression of the UPR sensors. This causes their activation and consequent response to ER stress. Grp78 increase is thus considered an upstream ER stress marker.

The transcription factor C/EBP homologous protein (CHOP) is another protein that is strictly correlated with ER stress. This factor is activated downstream, following ER signaling pathways mediated by the UPR sensors [21]. CHOP has been shown to be involved in ER stress-induced apoptosis [22] even if more recent findings indicate that CHOP can also activate genes involved in the regulation of the autophagic process [23].

Autophagy is a highly conserved metabolic process that involves the sequestration of cytoplasmic components, cellular proteins and organelles into the autophagosome, promotes cargo degradation and the recycling of cargo components. During the autophagosome assembly and maturation, LC3 and p62 proteins play a key role. LC3-I is converted into LC3-II, which directly participates in autophagosome formation and elongation and is considered an important autophagy marker. P62, apart from many other regulatory functions including Nrf2 regulation, takes part in autophagy delivering polyubiquitinated cargoes to the autophagosome for degradation [24,25].

This paper describes the involvement of oxidative stress, ER stress and autophagy in the cell death mechanism activated by tributyltin (IV) ferulate (TBT-F) in colon cancer cells. TBT-F is a novel organotin ferulic acid derivative that was synthesized in our laboratory with the purpose of increasing the anti-tumor efficacy of ferulic acid. In our previous paper, we described the chemical characterization of the compound and showed its anti-tumor effects in three colon cancer cell lines (HCT116, HT29 and Caco-2) [26]. Here, we provide evidence for the biochemical mechanism activated by TBT-F in colon cancer HCT116 cells, which were chosen as the most sensitive cell line to the effects of the compound among those we considered in our previous study. In particular, the results strongly support that the organotin compound exerts a pro-oxidant action, which causes ER-stress and autophagy that are related with colon cancer cell death.

## 2. Results

### 2.1. The Cytotoxic Effects of TBT-F Are Dependent on ROS Production

Our previous paper demonstrated that TBT-F, a novel synthetic compound that conjugates ferulic acid with tributyltin (IV), specifically induces autophagic cell death in different colon cancer cell lines without involving other forms of cell death, such as apoptosis or necroptosis [26]. To elucidate the molecular mechanism of TBT-F action, we first focused on a possible induction of oxidative stress. Therefore, in this study, we measured the levels of reactive oxygen species (ROS) under TBT-F treatment in HCT-116 cells, which resulted the most sensitive colon cancer cell line among those we previously investigated.

**Figure 1:**
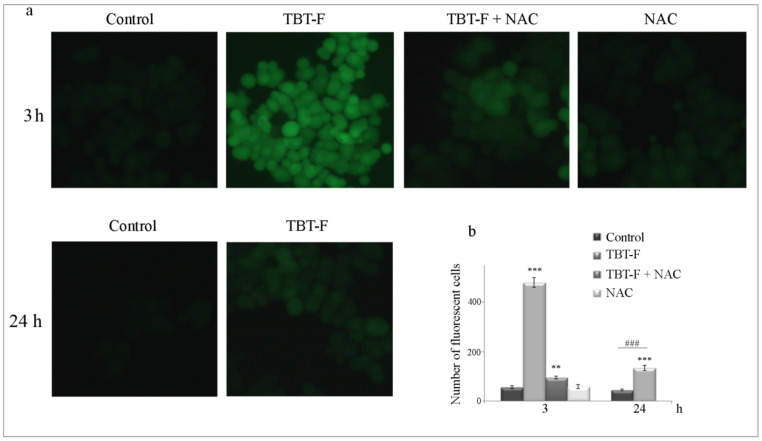
Tributyltin (IV) ferulate (TBT-F) stimulates intracellular reactive oxygen species (ROS) production. (**a**) HCT-116 cells (7 × 10^3^) were treated for 3 h with 400 nM TBT-F in either the presence or absence of 5 mM N-acetylcysteine (NAC) (upper panel) and for 24 h with TBT-F only (lower panel). The cells were then subjected to H_2_DCFDA staining as reported in Materials and Methods and visualized by fluorescence microscopy using fluorescein isothiocyanate (FITC) filter (magnification ×200). (**b**) The fluorescence was quantified by a Varian fluorescence spectrophotometer and values were shown as mean fluorescence intensity. The data are representative of three independent experiments with similar results. ** *p* < 0.01, *** *p* < 0.001 compared to the untreated sample; ^###^ *p* < 0.001 compared to TBT-F-treated sample at 3 h.

Based on our previously published dose-dependent results [26], in the experiments reported in this paper, we used the concentration 400 nM of TBT-F. As shown in Figure 1a, TBT-F induced a precocious and dramatic ROS production, clearly visible after 3-h treatment, as revealed by oxidized fluorochrome H_2_DCFDA (green fluorescence), a dye employed as a general indicator of cellular ROS. This effect was consistently attenuated by the addition of 5 mM antioxidant N-acetylcysteine (NAC). As shown in the same figure, prolonging TBT-F treatment time up to 24 h determined a significant reduction of ROS, which were just weakly detectable. Quantitative evaluation of ROS production under the same treatment conditions confirmed microscopic data (Figure 1b). 

**Figure 2:**
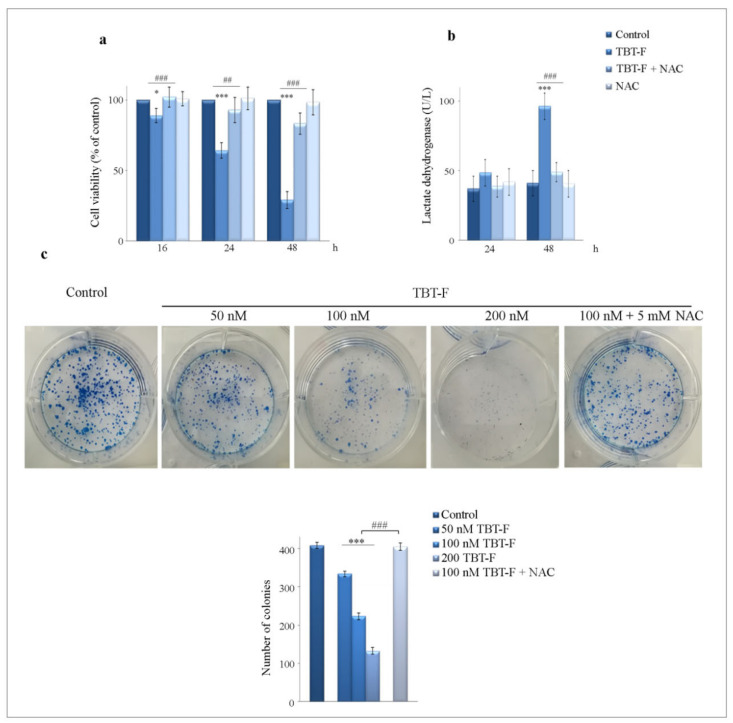
The antitumor effects of TBT-F and the influence of NAC. For the evaluation of cell viability (**a**) and lactate dehydrogenase release (**b**), cells (7 × 10^3^) were exposed to 400 mM TBT-F for the indicated times in either the presence or absence of 5 mM NAC. Cell viability was assessed by MTT assay and lactate dehydrogenase release by the Clinic Biochemistry Architect kit as reported in Materials and Methods. For clonogenic assay (**c**), single cell suspension (300 cells) was seeded in 6-well plates and after 48 h was treated with increasing doses of TBT-F. An amount of 5 mM NAC was only added to 100 nM FBT-F. Clonogenic assay was carried out after seven days and the number of colonies was counted as reported in Materials and Methods. The results reported in the histograms and the images are representative of three separate experiments. * *p* < 0.05, *** *p* < 0.001 compared to the untreated sample; ^##^ *p* < 0.01, ^###^ *p* < 0.001 compared to TBT-F-treated sample.

The influence of the antioxidant NAC on TBT-F effects was also evaluated by measuring cell viability, lactate dehydrogenase release and clonogenic ability of colon cancer cells. TBT-F reduced cell viability in a time-dependent manner (Figure 2a). The decreasing effect appeared at 16 h and reached the maximum at 48 h of treatment (−71%). Interestingly, at each time point, the addition of NAC almost completely prevented TBT-F effect on cell viability. In particular, at 48 h treatment in the presence of NAC the percentage of cell viability reduction was only 17%.

In order to assess whether the reduction in cell viability correlates with cell death, we measured lactate dehydrogenase release into the culture medium, which resulted significantly increased after 48 h treatment (96 U/L in treated cells versus 41 U/L in controls). Also in this case, NAC significantly prevented TBT-F effect (49 U/L) (Figure 2b).

In addition, we evaluated whether TBT-F was capable of influencing the clonogenic growth of colon cancer cells in either the presence or absence of NAC. As shown in Figure 2c, cells treated for seven days with increasing doses of TBT-F (50–200 nM) showed a significant dose-dependent inhibition of colony formation compared to the untreated control. Interestingly, in the presence of NAC the anti-clonogenic effect of TBT-F was completely prevented.

### 2.2. The Role of the Protective Factor Nrf2 and the Effect of Nrf2 Silencing

**Figure 3:**
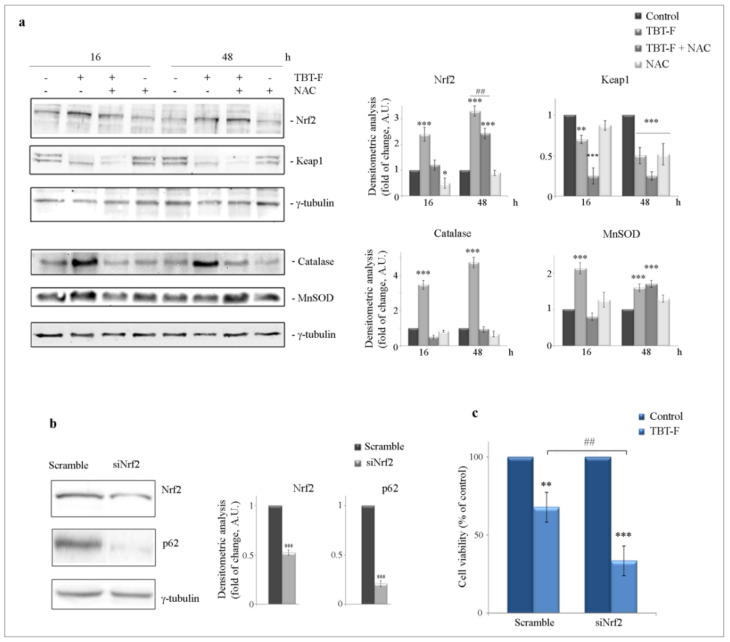
TBT-F-induced antioxidant response and effects of nuclear factor erythroid-2 related factor 2 (Nrf2) silencing. (**a**) Cells were treated for the indicated times with 400 nM TBT-F in either the presence or absence of 5 mM NAC. Then protein extracts were prepared and subjected to Western blotting analysis to determine the levels of Nrf2, Keap1 and antioxidant enzymes catalase and superoxide dismutase 2 (MnSOD). The correct protein loading was ascertained by immunoblotting for γ-tubulin. Representative blots of three independent experiments and densitometric analysis histograms are shown. * *p* < 0.05, ** *p* < 0.01, *** *p* < 0.001 compared to the untreated sample; ^##^ *p* < 0.01, compared to TBT-F-treated sample. (**b**) Nrf2 siRNA transfection was carried out as reported in Materials and Methods. SiNrf2 and scramble control transfected cells were subjected to Western blot analysis to verify Nrf2 silencing and the levels of p62. *** *p* < 0.001 compared to the untreated sample. (**c**) MTT assay was performed after treating Nrf2-silenced cells with 400 nM TBT-F for 24 h. Histograms shown are representative of three independent experiments. ** *p* < 0.01, *** *p* < 0.001 compared to the untreated sample; ^##^ *p* < 0.01 compared to TBT-F-treated sample.

Since it is well known that cells respond to ROS increase activating an antioxidant response, we analyzed the levels of Nrf2, its inhibitor Keap1 and the antioxidant enzymes catalase and superoxide dismutase 2 (MnSOD) by Western blot. As shown in Figure 3a, TBT-F induced a marked increase in the levels of Nrf2 at both 16- and 48-h treatment times. Concomitantly, TBT-F dramatically reduced the levels of Keap1, an effect that was already evident at 16-h treatment and maintained at 48 h. Interestingly, NAC markedly prevented the effect of TBT-F on Nrf2 at 16 h and attenuated it at 48 h. Surprisingly, the addition of NAC did not counteract the TBT-F reducing effect on Keap1, but rather increased it.

The evaluation of the effects on antioxidant enzymes showed that TBT-F treatment increased catalase and MnSOD levels, with a much higher effect on catalase. Also in this case, TBT-F effects were prevented by the addition of NAC. In particular, NAC completely abolished the effect on catalase expression at both treatment times and the effect on MnSOD only at 16 h (Figure 3a).

To clarify Nrf2 function in colon cancer cells in response to TBT-F, we silenced Nrf2 by RNA interference. As shown in Figure 3b, a marked reduction in Nrf2 level was found in Nrf2 siRNA transfected cells compared to scramble control. The same blot shows that the level of p62 was dramatically decreased in Nrf2-silenced cells, thus supporting the hypothesis that Nrf2 can activate p62 gene transcription as demonstrated by Jain et al. [27].

Interestingly, Nrf2 targeting also increased the cytotoxic effect of TBT-F. In Nrf2-silenced cells, indeed, cell viability reduction induced by the compound anticipated at 24 h, as revealed by morphological analysis (not shown) and MTT assay (−67% compared to −36% referred to scramble control transfected cells) (Figure 3c).

### 2.3. TBT-F Induces ER Stress Markers and NAC Prevents These Effects

**Figure 4:**
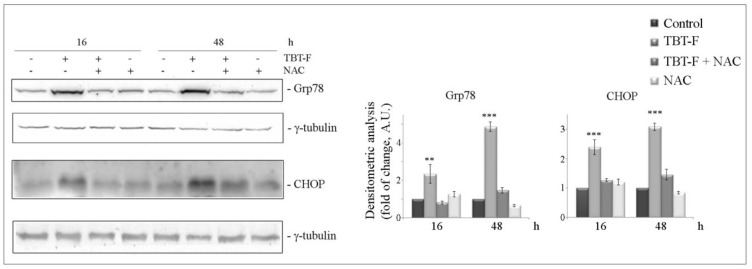
The effect of TBT-F on endoplasmic reticulum (ER) stress markers. Cells were treated for the indicated times with 400 nM TBT-F in either the presence or absence of 5 mM NAC, then harvested and protein extracts were prepared and subjected to Western blotting analysis to determine the levels of glucose regulated protein (Grp78) and C/EBP homologous protein (CHOP). The correct protein loading was ascertained by immunoblotting for γ-tubulin. Representative blots of three independent experiments and densitometric analysis histograms are shown. ** *p* < 0.01, *** *p* < 0.001 compared to the untreated sample.

Considering the mutual connection between ROS and ER stress induction and the rapid ROS production in colon cancer cells following TBT-F treatment, we explored the effect of the compound on ER stress components and a possible relationship with oxidative stress [28]. To this purpose, we analyzed, by Western blot, the levels of two important markers, the upstream ER stress chaperone Grp78 and the downstream ER stress transcription factor CHOP under TBT-F treatment in either the presence or absence of NAC. The results showed that TBT-F markedly increased the levels of the ER stress markers, an effect that was clearly visible at 16 h treatment and further increased at 48 h (Figure 4). It is interesting to note that at each time point the addition of the antioxidant NAC completely prevented the effect of TBT-F on both Grp78 and CHOP levels. Overall, the effects induced by TBT-F on the two ER stress markers and the reversion induced by NAC strongly suggest that ROS production is responsible for the triggering of an ER stress response in our model.

### 2.4. TBT-F Activates ROS-Dependent Autophagy Correlated with ER Stress

**Figure 5:**
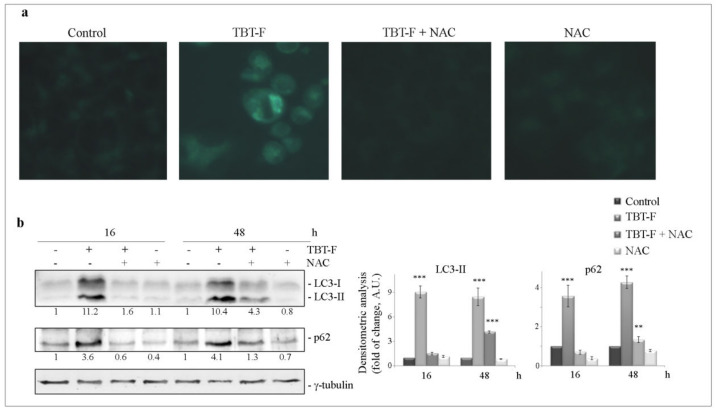
Autophagic effects of TBT-F. (**a**) Intracellular vacuolization evidenced by monodansylcadaverine (MDC) staining. Cells (7 × 10^3^) were treated for 16 h with 400 nM TBT-F in either the presence or absence of 5 mM NAC. Afterward, cells were incubated with 50 μM MDC for 10 min, washed with PBS and visualized under a fluorescence microscope equipped with a DAPI filter at a magnification of ×400. Micrographs are representative of almost three fields from two independent experiments. (**b**) Western blot analysis of autophagic markers. Cells were treated for the indicated times with 400 nM TBT-F in either the presence or absence of 5 mM NAC, then protein extracts were subjected to Western blotting analysis to determine the levels of LC3 and p62. The correct protein loading was ascertained by immunoblotting for γ-tubulin. Representative blots of three independent experiments and densitometric analysis histograms are shown. ** *p* < 0.01, *** *p* < 0.001 compared to the untreated sample.

Finally, we investigated whether ROS production and consequent ER stress induction could be related with TBT-F-induced autophagic cell death that we previously demonstrated in colon cancer cells [26]. Therefore, we first evaluated the effect of NAC on the formation of autophagic vacuoles in TBT-F-treated cells. As shown in Figure 5a, monodansylcadaverine (MDC) staining evidenced that NAC completely abolished green fluorescent dot-like structures (corresponding to autophagic vacuoles) observed under TBT-F treatment. As a confirmation, we also evaluated the effect of NAC on autophagic proteins. In accordance with our previous paper, the results showed that TBT-F increased the levels of both LC3-II and p62 autophagic markers in time-dependent manner. The effect appeared at 16 h of treatment and was maintained at 48 h. In both conditions, NAC consistently prevented the effect of TBT-F (Figure 5b).

**Figure 6:**
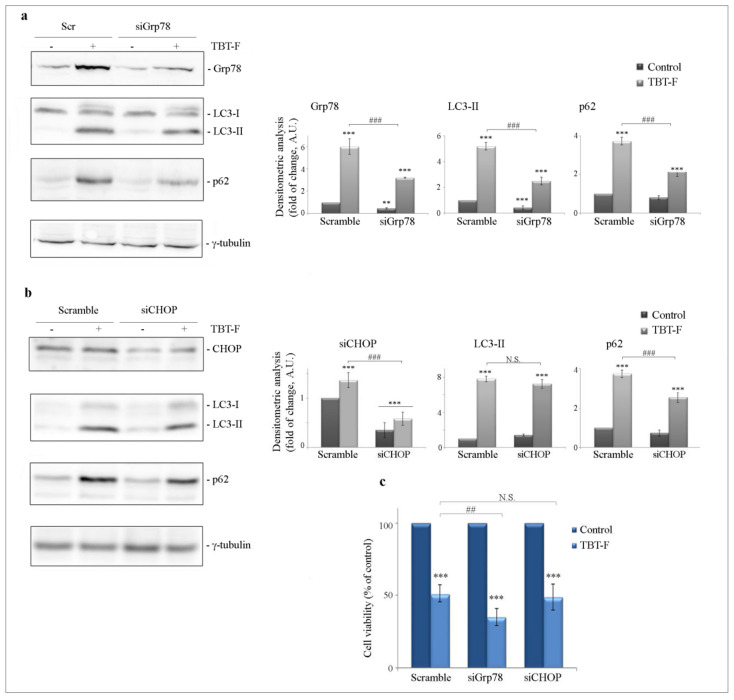
The effects of ER stress markers silencing on autophagic markers and cell viability. Grp78 (siGrp78) and CHOP (siCHOP) transfections were carried out as reported in Materials and Methods. Grp78-silenced cells (**a**), and CHOP-silenced cells (**b**), together with scramble controls, were treated with 400 nM TBT-F for 24 h and subjected to Western blot analysis. The silencing efficiency was determined using Grp78 and CHOP-specific antibodies. The effects of silencing on autophagic LC3 and p62 proteins were then evaluated. The correct protein loading was ascertained by immunoblotting for γ-tubulin. Representative blots of three independent experiments and densitometric analyses are shown in the histograms. ** *p* < 0.01, *** *p* < 0.001, compared to the untreated sample; ^###^ *p* < 0.001 compared to scramble TBT-F-treated sample. (**c**) MTT assay was performed after treating transfected cells with TBT-F for 24 h. All the histograms shown are representative of three independent experiments. *** *p* < 0.001, compared to the untreated sample; ^##^ *p* < 0.01 compared to scramble TBT-F-treated sample; N.S.—not significant.

Many lines of evidence indicate that ER stress and autophagy can be interdependently connected [29]. In an attempt to elucidate the relationship between ROS-dependent ER stress and autophagy in our model, we silenced Grp78 and CHOP proteins by RNA interference and then evaluated the effects on autophagic markers. The silencing efficiency was confirmed in both cases by Western blot analysis (Figure 6a,b). As shown in Figure 6a, the levels of both LC3-II and p62 were consistently decreased in Grp78-silenced cells following TBT-F treatment compared to TBT-F-treated scramble control. On the other hand, CHOP silencing did not seem to influence TBT-F effect on LC3-II but slightly decreased the effect on p62.

Interestingly, we found that Grp78 silencing increased by about 25% the cytotoxic effect of TBT-F compared to control (Figure 6c), whereas CHOP silencing did not produce significant effects on cell viability following TBT-F treatment.

## 3. Discussion

Tributyltin(IV) ferulate (TBT-F) has been synthesized with the purpose to improve the anti-tumor effects of ferulic acid (4-hydroxy-3-methoxy cinnamic acid), a natural compound with antioxidant activity [30,31].

It is well known that organotins(IV) induce tissue damage which is prevalently associated with the induction of oxidative stress [32,33,34,35]. On the other hand, ferulic acid is a phenolic compound, characterized by an aromatic ring carrying hydroxyl groups (free or substituted) as substituents, and is known to exhibit strong effects on scavenging free radicals and antioxidant activity by donating hydrogen atoms or electrons [31]. Although our previous study [26] demonstrated that ferulic acid does not use the phenolic hydroxyl group to coordinate organotin (IV) moiety, here, we demonstrate that in coordination with organotin (IV), it acquires pro-oxidant properties. It is possible to hypothesize that in the coordinated molecule pro-oxidant properties of organotin (IV) moiety prevail on the supposed antioxidant action of the ferulic acid component. Our results, indeed, showed that the compound dramatically increases intracellular ROS production in colon cancer HCT116 cells, an effect that was completely prevented by the addition of the general antioxidant NAC. Studies are in progress to verify the sources of ROS. Our unpublished results seem to exclude NADPH oxidase, an enzyme that is involved in cytosolic generation of superoxide anion [36], because its inhibitor apocynin was not capable of preventing the TBT-F effect. We, thus, hypothesize that the precocious ROS rise derives from other sources, most likely from mitochondria, which represent the primary origin of intracellular ROS [37].

The precocious ROS burst seems to be sufficient to account for the cytotoxic effects of TBT-F as evidenced by the protective action of NAC on cell viability decrease, lactate dehydrogenase release and anti-clonogenic growth induced by the compound. At the same time, due to ROS increase, cells activated the defensive antioxidant Nrf2/Keap1 axis, as revealed by the remarkable increase in the levels of Nrf2 and the antioxidant enzymes catalase and MnSOD with the concomitant reduction of the Nrf2 negative regulator Keap1.

The observation that NAC completely prevented Nrf2 increase suggests that ROS account for the stimulation of Nrf2-mediated antioxidant response. This finding is in line with the results obtained by Zhou et al., who showed that ROS production induced by cisplatin exposure in Hep-2 cells is regulated by Nrf2 and reversed by the addition of the antioxidant NAC [38]. In accordance, in our model NAC also prevented the increase in the levels of the antioxidant enzymes catalase and MnSOD. On the other hand, the reason why NAC was not capable of counteracting the decrease in Keap1 induced by TBT-F, but it rather seemed to promote this decreasing effect, remains to be elucidated. A speculative interpretation can be that TBT-F can directly interact with Keap1 thus promoting its degradation independently of ROS production. Keap1 is a sensor protein containing nucleophilic groups such as sulfidrilic cysteine residues that can directly bind electrophilic groups present in TBT-F. The electron acceptor properties of organotin (IV) compounds could favor their interaction with electron donors atoms such as sulfur in biomolecules. It is known, indeed, that many mechanisms of organotin (IV) toxicity are due to the ability of these compounds to form covalent adducts with cellular proteins via reactive cysteine residues [39]. Covalent interactions between organotins (IV) and reactive cysteines residues of peptide stannin, retinoid receptor α and CYP450 enzymes, such as aromatase, have been described [40]. Usually adduct formation perturbs the molecular organization within the cells, altering protein structure and function, thereby promoting cell death pathways [41]. Ongoing studies aim to evaluate these hypotheses.

Considering the key role of oxidative stress in cell death induced by TBT-F, our results show that targeting Nrf2 can be a valuable tool to increase the antitumor potency of the compound. The cytotoxic effect of TBT-F increased, indeed, in Nrf2-silenced cells. In addition, our results show that Nrf2 interference produced a dramatic fall in p62 levels. Accordingly, it has been proposed that the interplay between p62 and Nrf2 is mutual. On one hand, sequestering Keap1 by p62 leads to Nrf2 activation, on the other, p62 has been demonstrated to be an Nrf2 transcriptional target [42].

It has been widely documented that oxidative stress can promote ER stress with the consequent unfolded protein response (UPR) as well as autophagy induction [43]. Interplay among these processes involves a complex network of interactions. ER stress and autophagy can mutually regulate each other. For instance, autophagy is activated following an ER stress condition in neurons to alleviate and protect against neurodegeneration [44]. In other conditions, instead, the induction of autophagy following ER stress triggers type II programmed cell death [45].

Data reported in this paper show that TBT-F increased the levels of ER markers Grp78 and CHOP and, at the same time, induced autophagy, as shown by MDC positivity and remarkable increase in the levels of LC3-II and p62 autophagic markers. Although the level of p62 usually decreases following degradation by the autophagosome when autophagy is completed, in our model we found accumulation of p62 even at a prolonged treatment time (48 h). We could interpret these data as the consequence of p62 induction by TBT-F, which could be sustained by Nrf2 as we demonstrated, or other p62 transcriptional activators.

It is interesting to note that the increase in both LC3-II and p62 levels were completely prevented by the antioxidant NAC. This observation prompted us to consider that both ER stress and autophagy are a consequence of TBT-F-induced ROS production and that ER stress most likely precedes autophagy induction as suggested by Grp78 silencing, which affected the levels of both p62 and LC3-II. These results strongly suggest that Grp78 is somehow necessary for autophagy triggering, a finding which is in line with the observation that Grp78 knockdown reduced autophagic proteins in neural cells [46]. Considering that Grp78 silencing can favor ER stress as suggested by other authors [47,48], here we show that Grp78 silencing slightly increased the cytotoxic effect of TBT-F. In this case, it is possible to speculate that ER stress prevails over autophagy. Moreover, our unpublished data suggest that stimulating ER stress by proteasome inhibition increases the cytotoxic effect of TBT-F switching autophagic cell death into apoptotic cell death.

On the other hand, CHOP silencing did not produce significant effects on cell viability and autophagic LC3-II while it slightly decreased the TBT-F effect on p62. Based on our results, we can interpret that CHOP silencing did not affect cell viability because this factor is mainly involved in apoptosis, which we did not observe under TBT-F treatment. However, we cannot completely exclude that CHOP can be also related to autophagy. The effects observed on p62 are in accordance with B’Chir et al. who have shown that CHOP can transcriptionally activate SQSTM1 gene encoding p62 protein [49]. Moreover, data present in the literature indicate that it may favour the expression of other autophagic genes [50]. Nevertheless, it has to be considered that interconnection between ER stress and autophagy can depend on other upstream ER stress-related UPR components, such as ATF4, which is activated following protein kinase R-like endoplasmic reticulum kinase (PERK) signaling [51].

**Figure 7:**
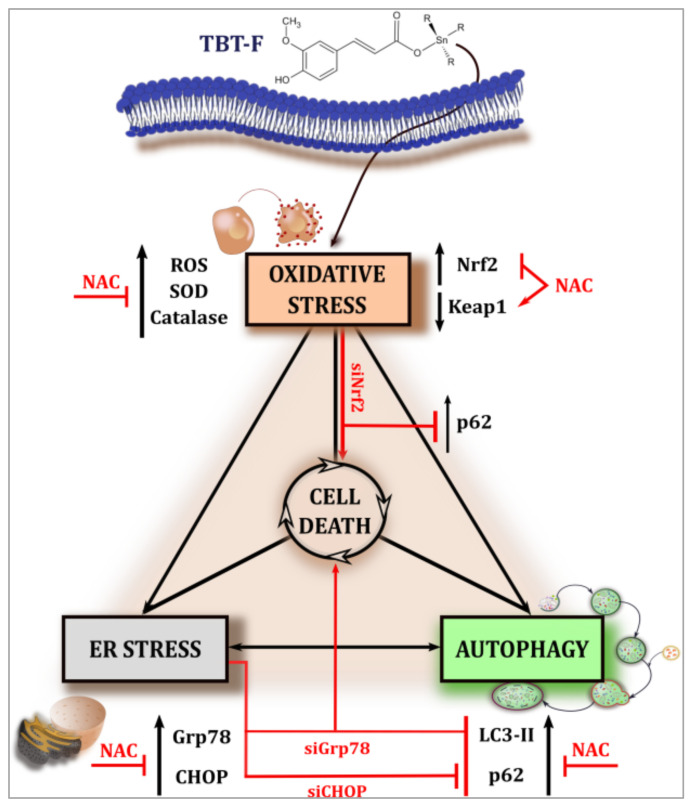
Schematic representation of the mechanism induced by TBT-F.

Taken together, the results presented in this paper strongly suggest that TBT-F acts as a pro-oxidant compound in colon cancer cells and its action mechanism specifically involves ROS/ER stress/autophagy interplay as summarized in Figure 7. In particular, ROS and ER stress are most likely responsible for the activation of autophagy that we interpreted as a cell death process. This hypothesis is supported by our previous findings that autophagy inhibition markedly prevented the cytotoxic effect of TBT-F and that other forms of cell death, such as apoptosis or necroptosis were not involved.

## 4. Materials and Methods

### 4.1. Chemicals and Reagents

Tributyltin (IV) ferulate (TBT-F) compound was synthetized in our laboratory as previously described [26]. For in vitro experiments, TBT-F was dissolved in DMSO (10 mM stock solution). Prior to use, stock solution was opportunely diluted in DMEM culture medium, not exceeding 0.04% (*v*/*v*) DMSO, to realize the proper TBT-F final concentration. Equal volumes of DMSO were added to untreated cells as vehicle control. N-acetylcysteine (NAC) was purchased from Sigma (Milan, MI, Italy), and dissolved in DMEM culture medium (200 mM stock solution). The pH was then adjusted to 7.4. 5 mM NAC (final concentration) was used to treat the cells. This concentration was chosen as the proper one to obtain protective effects with respect to oxidative stress without causing toxic effects to the cells, according to our previous dose-dependent evaluation. DMSO was also added as vehicle control to NAC-treated samples.

All materials for cell cultures were purchased from Euroclone (Pero, Italy), and Life Technologies Ltd. (Monza, Italy).

### 4.2. Cell Culture Conditions

HCT116 colon cancer cells (American Type Culture Collection, Rockville, MD, USA) were cultured as previously described [9]. For the experiments, cells were plated at a density of 7 × 10^3^ or 1.5 × 10^5^/well in 96- or 6-well plates, respectively, and allowed to adhere overnight. Subsequently, cells were treated with the chemicals or vehicle only and the incubation was protracted for the established times.

### 4.3. Evaluation of Cell Viability

HCT116 cell viability was determined by 3-(4,5-dimethylthiazol-2-yl)-2,5-diphenyltetrazolium bromide (MTT) assay, as previously described [52]. Values reported in figures are expressed as percentage of the viability of treated cells compared with untreated control (100% viability). The experiments were performed in triplicate and data are shown as mean ± SD of three independent experiments.

### 4.4. Lactate Dehydrogenase Release Assay

Lactate dehydrogenase (LDH) release assay assesses plasma membrane increased permeability when cells are subjected to membrane damages, monitoring the release into the cell culture medium of the stable cytosolic enzyme lactate dehydrogenase (LDH). Cells (7 × 10^3^) were plated in 96-well plates, and after 24 h, culture medium was refreshed with 100 µL phenol red-free DMEM and treatments were performed. After treatments, cells were recovered and LDH concentration evaluated by Abbot Clinic Biochemistry Architect kit (Chicago, IL, USA).

### 4.5. Clonogenic Assay

Clonogenic assay allows detecting the ability of isolated cells to form clones and verify the effect of a given compound on the clonogenic growth. According to previous protocol [53], HCT116 cells were plated as single cells in 6-well tissue culture plates (300 cells/well), and incubated at 37 °C. After 2 days from seeding, cells were treated with TBT-F for 7 days and the medium was replaced at day 4. Colonies were then fixed and stained with 1% (*v*/*v*) methylene blue solution dissolved in 50% PBS/ethanol for 1 min at room temperature. Then, colonies were air-dried, observed at the light microscope (Leica DMR, Microsystems S.r.l, Wetzlar, Germany) and counted. For counting, each well was divided into four quadrants and the media of the number of clones in each quadrant was estimated. Then the total number per well was deduced. Only clones containing more than 50 cells were considered.

### 4.6. Intracellular ROS Measurement

Intracellular ROS production was assessed by 2′,7′ dichlorodihydrofluorescein diacetate (H_2_DCFDA) (Molecular Probes, Eugene, OR, USA) staining, as previously reported [54]. HCT116 cells (7 × 10^3^ cells/well) were seeded in 96-well plates and incubated with TBT-F (400 nM) for 3 or 24 h. After treatment, cells were washed with PBS, containing 5 mM glucose and stained with H_2_DCFDA (20 µM final concentration) at 37 °C for 30 min in the dark. Cells were then washed with PBS and H_2_DCFDA-positive cells were analyzed by fluorescence microscopy using an appropriate fluorescein isothiocyanate (FITC) filter (Leica DMR, Microsystems S.r.l, Wetzlar, Germany). All images were acquired by a computer imaging system (Leica DC300F camera) and three different visual fields were examined for each condition. The fluorescence intensity was estimated by a Varian CARY Eclipse Fluorescence Spectrophotometer (Varian Medical Systems Italia SpA, Milan, Italy). Data reported represent the means of three independent experiments. ROS generation was measured in a fluorescent plate reader (excitation at 488 nm and emission at 520 nm). Cells incubated with 10 µM H_2_O_2_ were used as a positive control (*n* = 9 per condition).

### 4.7. Monodansylcadaverine Labeling

Autophagy was detected by staining autophagic vacuoles with monodansylcadaverine (MDC) as previously described [9]. HCT116 cells (7 × 10^3^ cells/well) were plated in 96-well plates and treated for the indicated times. After treatments, cells were stained with 0.05 mM MDC in PBS at 37 °C for 10 min in the dark. Cells were then washed with PBS and analyzed under a Leica DMR fluorescence microscope (Wetzlar, Germany) equipped with a 4′,6-diamidino-2-phenylindole (DAPI) filter system. Pictures were acquired by a computer imaging system (Leica DC300F camera) and three different visual fields were examined for each condition.

### 4.8. Western Blot Analysis

After treatments, whole-cell extracts were prepared in ice-cold lysis RIPA buffer (1% NP-40, 0.5% sodium deoxycholate and 0.1% SDS in PBS, pH 7.4), supplemented with a protease inhibitor cocktail, and subjected to Western blot as previously reported [55]. In the experiments, the correct protein loading was verified by both Ponceau red staining and housekeeping protein γ-tubulin immunodetection. Specific primary antibodies directed against CHOP, Grp78, SOD, Catalase, Keap1 (diluted 1:500), were purchased from Santa Cruz Biotechonology (St. Cruz, CA, USA); γ-tubulin and p62 (diluted 1:1000) from Sigma Aldrich (Milan, Italy); LC3 and Nrf2 (diluted 1:1000) from Novus Biologicals (Milano, Italy). The Immunodetection was carried out by electrochemical luminescence labeling system (ECL) using ChemiDoc, XR Image system (Bio-Rad Laboratories, Hercules, CA, USA). The intensity of the protein bands was quantified using Quantity One Imaging Software (Bio-Rad Laboratories) and reported as the ratio of the intensity of protein bands normalized to γ-tubulin, versus the intensity of the untreated samples. All the blots shown in figures are representative of three independent experiments.

### 4.9. Gene Silencing

Gene silencing experiments were performed using specific siRNAs targeting Nrf2 (30 nM for each siRNA, SI03246950 and SI03246614, Qiagen, Hilden, Germany), CHOP (100 nM, D-004819-02-0005, Dharmacon RNA Technologies, Chicago, IL, USA), Grp78 (50 nM, Santa Cruz Biotechnology, CA, USA) and non-silencing scramble siRNA (60 nM, SI03650318, Qiagen, Hilden, Germany). HCT116 cells (1.5 × 10^5^ cells/well) were plated into 6-well plates and allowed to adhere overnight to reach about 60% confluence. Specific siRNAs and negative siRNA control (non-silencing siRNA) were transfected for 6 h into the cells in the presence of 5 µL Lipofectamine 2000 (Invitrogen, Carlsbad, CA, USA) in a final volume of 1 mL serum/antibiotic-free DMEM medium supplemented with 2 mM glutamine. The reaction was stopped by replacing the culture medium with complete fresh DMEM. Twenty-four hours after siRNA transfection, silenced cells were treated with TBT-F (400 nM) for an additional 24 h (Nrf2, and CHOP silencing) or 48 h (Grp78 silencing).

### 4.10. Statistical Analysis

Data were represented as mean ± S.D. and analysis was performed using the Student’s *t*-test and one-way analysis of variance. Comparisons between the control (untreated) vs. all treated samples were made. Statistical significance was determined using one-way ANOVA followed by post-hoc Bonferroni’s test. The statistical significance threshold was fixed at *p* < 0.05.

## 5. Conclusions

The results provided in this study provide novel evidence that TBT-F behaves as a pro-oxidant drug in colon cancer cells stimulating ROS production and consequent ER stress and autophagy. Targeting cellular antioxidant response markedly increased the TBT-F action. In addition, our work reveals that ER stress and autophagy are strictly correlated with each other since inhibition of ER stress reduces autophagic marker levels. Therefore, TBT-F displays a cytotoxic action based on ROS/ER stress/autophagy triad that might be important in colon cancer, particularly when resistance to classic apoptosis occurs.
